# Single-cell transcriptome mapping identifies a local, innate B cell population driving chronic rejection after lung transplantation

**DOI:** 10.1172/jci.insight.156648

**Published:** 2022-09-22

**Authors:** Natalia F. Smirnova, Kent Riemondy, Marta Bueno, Susan Collins, Pavan Suresh, Xingan Wang, Kapil N. Patel, Carlyne Cool, Melanie Königshoff, Nirmal S. Sharma, Oliver Eickelberg

**Affiliations:** 1 Division of Pulmonary Sciences and Critical Care Medicine, Department of Medicine, University of Colorado Anschutz Medical Campus, Aurora, Colorado, USA.; 2 Institut des Maladies Métaboliques et Cardiovasculaires (I2MC) — INSERM U1297, University of Toulouse III, Toulouse, France.; 3 RNA Bioscience Initiative, University of Colorado Anschutz Medical Campus, Aurora, Colorado, USA.; 4 Division of Pulmonary, Allergy, and Critical Care Medicine, Department of Medicine, University of Pittsburgh, Pittsburgh, Pennsylvania, USA.; 5 Center for Advanced Lung Disease and Lung Transplantation, University of South Florida/Tampa General Hospital, Tampa, Florida, USA.; 6 Department of Pathology, University of Colorado Anschutz Medical Campus, Aurora, Colorado, USA.; 7 Division of Pulmonary & Critical Care, Brigham and Women’s Hospital, Harvard Medical School, Boston, Massachusetts, USA.

**Keywords:** Pulmonology, Transplantation, Organ transplantation

## Abstract

Bronchiolitis obliterans syndrome (BOS) is the main reason for poor outcomes after lung transplantation (LTx). We and others have recently identified B cells as major contributors to BOS after LTx. The extent of B cell heterogeneity and the relative contributions of B cell subpopulations to BOS, however, remain unclear. Here, we provide a comprehensive analysis of cell population changes and their gene expression patterns during chronic rejection after orthotopic LTx in mice. Of 11 major cell types, *Mzb1*-expressing plasma cells (PCs) were the most prominently increased population in BOS lungs. These findings were validated in 2 different cohorts of human BOS after LTx. A *Bhlhe41*, *Cxcr3*, and *Itgb1* triple-positive B cell subset, also expressing classical markers of the innate-like B-1 B cell population, served as the progenitor pool for *Mzb1*^+^ PCs. This subset accounted for the increase in IgG_2c_ production within BOS lung grafts. A genetic lack of Igs decreased BOS severity after LTx. In summary, we provide a detailed analysis of cell population changes during BOS. IgG^+^ PCs and their progenitors — an innate B cell subpopulation — are the major source of local Ab production and a significant contributor to BOS after LTx.

## Introduction

Chronic lung diseases are a leading cause of morbidity and mortality worldwide, accounting for 7% of all deaths from noncommunicable diseases (1). To date, lung transplantation (LTx) represents the only potential cure for most end-stage chronic lung diseases. Although short-term survival after LTx has significantly improved over time, the median survival and long-term survival of LTx are severely limited, primarily due to chronic rejection of the allograft, which leads to an irreversible decline of lung function. Adults who underwent primary LTx in the recent era (2010 to June 2017) had a median survival of 6.7 years, the worst of any solid-organ transplantation (2). The long-term outcome of LTx is mainly limited by the development of bronchiolitis obliterans syndrome (BOS), the most common phenotype of chronic rejection. BOS develops in 50% of all patients 5 years after LTx and is the leading cause of death after receiving a lung transplant (2). During the development of BOS, airways are progressively infiltrated by leukocytes and exhibit signs of chronic inflammatory and immune processes, ultimately resulting in peribronchial fibrosis and the loss of the bronchial epithelium. This scarring, due to epithelial loss, leads to an obstruction of the airways, decreasing the diameter of their lumen, limiting airflow, and reducing lung function (3).

We and others recently reported that B cells are a major contributing cell type to the development of BOS (4, 5), which has also been reported in chronic rejection of other solid organs after transplantation (6). Most of these studies have characterized systemic or local B cells in chronic rejection, using flow cytometry (7) or immunostaining of tissue sections (4, 5), introducing an intrinsic bias due the use of prespecified surface markers. The advent and validation of new methods, such as single-cell RNA-Seq (scRNA-Seq), offer the possibility to avoid this bias by distinguishing cell populations on the basis of an entire set of transcripts expressed by each cell. The scRNA-Seq technology has already changed preexisting classifications of immune cell subsets (8) and provided a new level of understanding of their distribution and function in disease (9, 10).

Here, we sought to provide a comprehensive analysis of cell population changes of BOS after LTx using scRNA-Seq to identify changes in the composition of cell types present during rejection inside the lung grafts and alternations to their individual gene expression profiles. Plasma cells (PCs) were the most enriched cell cluster detected in BOS. PCs were consistently found in large amounts in subepithelial layers of BOS airways, both in human disease and mouse models thereof. Detailed subclassification analysis of B cells and their progeny in murine lung grafts identified a subset of B cells that give rise to PCs. These B cells expressed a number of markers of the innate B-1 cell population and were capable of producing IgG ex vivo. Mechanistic interrogation of these findings in vivo revealed that a mouse strain lacking production of all but IgM Ig isotypes (*Aicda^–/–^* mice) displayed protection against the development of BOS after LTx.

## Results

### scRNA-Seq identifies BOS-associated cell populations.

**Figure 1:**
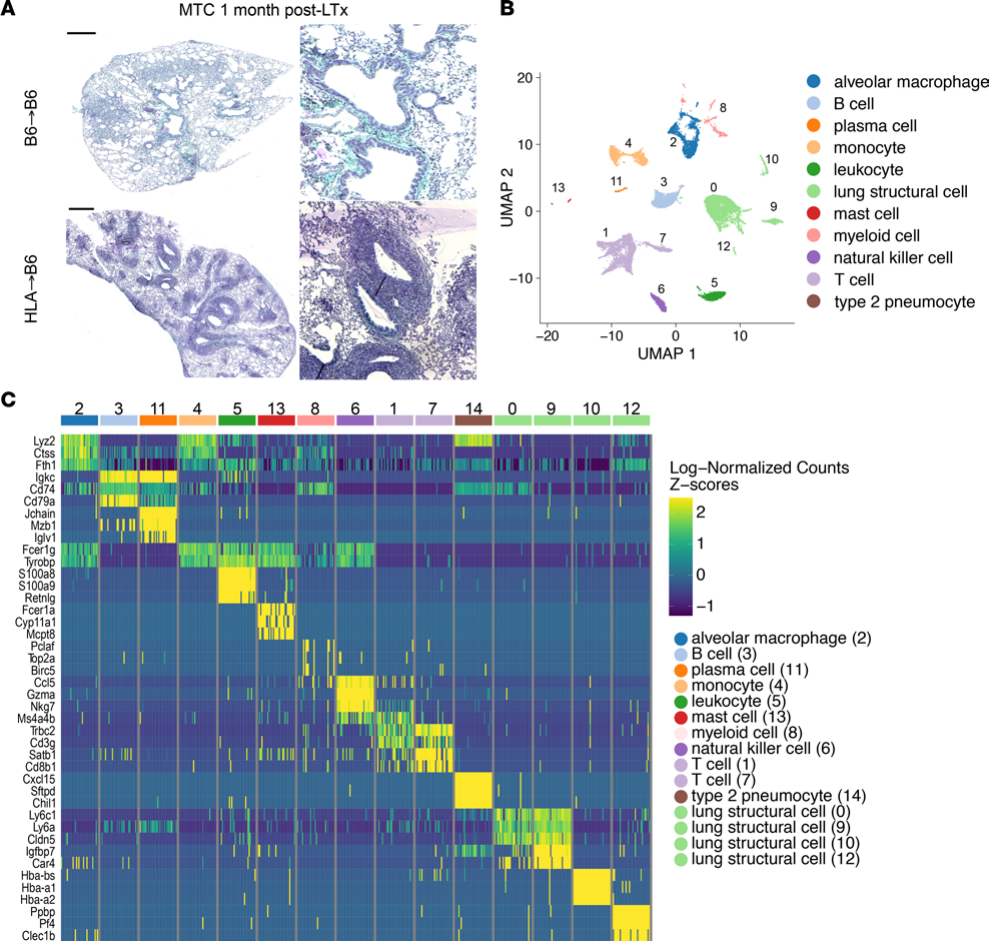
scRNA-Seq identifies 11 cell populations in mouse lung grafts. (**A**) Representative histology sections (Masson’s trichrome [MTC] staining) of control (B6→B6) and BOS (HLA→B6) mouse lung grafts 1 month after LTx. Scale bars, 500 μm. High magnification inserts are presented on the right. Original magnification, ×4. (**B**) UMAP representation of 11 distinct cell populations from unsupervised clustering of gene expression data in single cells extracted from control (B6→B6) and BOS (HLA→B6) transplanted mouse lungs. (**C**) Heatmap showing most upregulated genes for each of the cell clusters.

**Figure 2:**
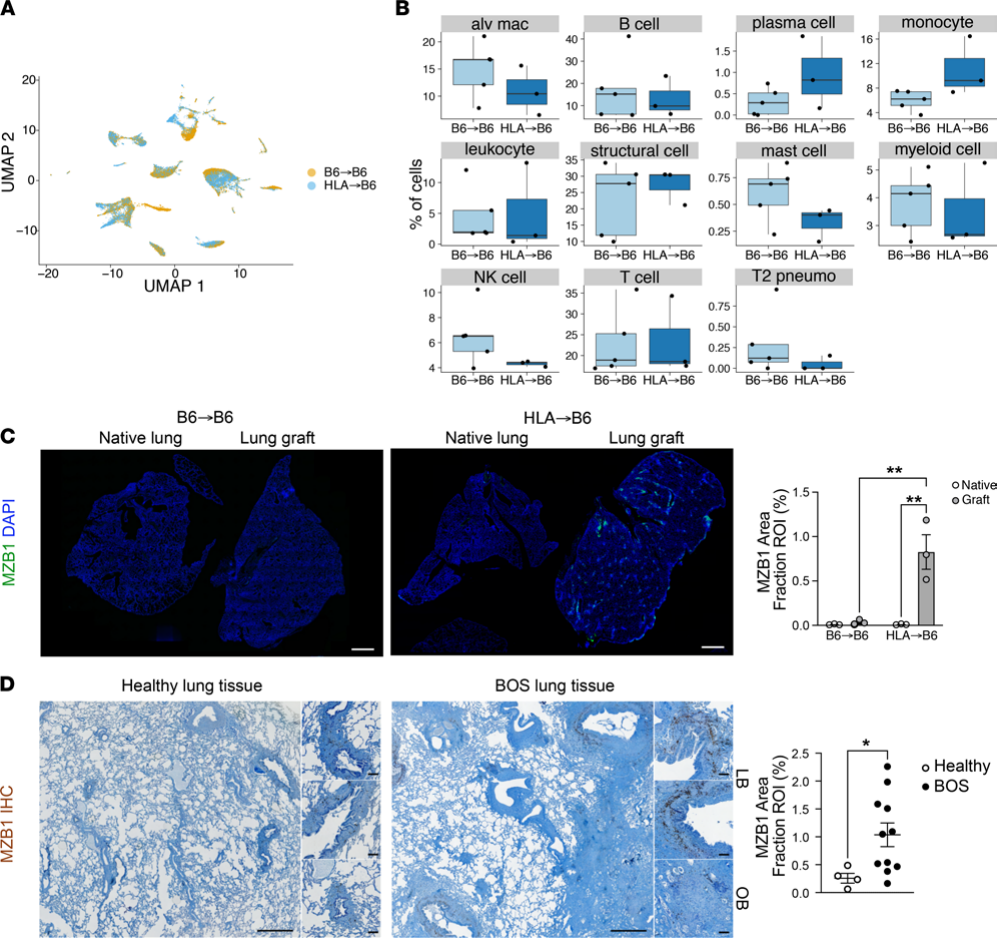
MZB1^+^ cells are associated with BOS in mouse and human transplanted lungs. (**A**) UMAP representation of the cell populations detected in murine lung grafts by condition (HLA→B6 and B6→B6). (**B**) Proportion of each cell type in control versus BOS mouse lung graft samples. (**C**) Immunofluorescence and quantification of Mzb1^+^ cells in mouse native lungs and lung grafts. Data analyzed with 2-way ANOVA and Tukey’s multiple comparisons posttest. Scale bars, 1 mm. (**D**) IHC and quantification for MZB1 on explants from healthy donors and patients who developed BOS after LTx (samples from the University of Colorado collection). Scale bars, 2 mm and 200 μm in inserts. Data are expressed as mean ± SEM and analyzed with a Mann-Whitney 2-tailed test. *P < 0.05, **P < 0.01; alv, alveolar; mac, macrophages; OB, obliterative bronchiolitis; pneumo, pneumocytes; ROI, region of interest; LB, lymphocytic bronchiolitis.

C57BL/6 (B6) recipient mice underwent orthotopic left LTx using lungs from B6 (control) of HLA (chronic rejection) donors (syngeneic group: B6→B6, *n* = 5; and BOS group: HLA→B6, *n* = 3), as previously described (4), to generate robust lymphocytic bronchiolitis (LB) and BOS. Lung grafts were harvested at 1 month after LTx (Figure 1A), a time point representing progression toward LB/BOS (11). Lung grafts were dissociated using a combination of enzymatic and mechanical methods, after which total cells were analyzed by scRNA-Seq. After application of quality-control filters and removal of ambient RNA, 9697 cells from syngeneic grafts and 11,081 cells from BOS grafts were included in scRNA-Seq analysis (detailed composition of cells per sample is shown in Supplemental Figure 1A; supplemental material available online with this article; https://doi.org/10.1172/jci.insight.156648DS1). Cellular gene expression data from both conditions were aligned and projected in a bidimensional uniform manifold approximation and projection (UMAP) plot to identify cell populations present in the lung grafts. Unsupervised clustering, using the *Seurat* package, distinguished 14 distinct cell clusters on the basis of their individual gene expression profiles (Figure 1, B and C). Putative biological identities were assigned to each cluster, using patterns of established canonical markers as well as importing the murine gene expression atlas, which resulted in the identification of 11 cell types (12) (Supplemental Figure 1, B–D). As shown in Figure 1B, lung grafts were composed of structural resident, as well as infiltrating or resident immune and inflammatory cells. Major shifts in cell abundance of each cluster were observed between the syngeneic and BOS groups (Figure 2, A and B). The most prominently increased cell type in chronic rejection were PCs, defined by the expression of *Xbp1*, *Prdm1* (encoding BLIMP-1), *Sdc1* (encoding CD138), and *Mzb1* (13, 14) (Figure 2, B and C). On the basis of this finding and on our previous data demonstrating a major role of B cells in the development of BOS (4), we next sought to define this cluster in more detail and interrogate the mechanistic contribution and clinical relevance of B cell subpopulations and PCs in BOS.

Of note, the resident structural cell compartment was subdivided into 11 distinct clusters, which we annotated manually (Supplemental Figure 2). Major quantitative shifts were observed for structural clusters 0 to 3. Cluster 0, enriched in BOS grafts, was identified as lymphatic endothelial cells, on the basis of their positivity for *Tmem100* and *Lyve1* (15). Interestingly, and consistent with its association with rejection, endothelial cells in this cluster were highly positive for genes encoding MHC II molecules. Expression of MHC II is enhanced on endothelial cells in inflammatory contexts, conferring them an antigen-presenting role (16). Consistent with the pathological changes documented for BOS (17, 18), cluster 1 and cluster 2 cells, expressing lung epithelial cell markers *Scgb1a1* and *Sftpc*, were decreased in proportion in rejecting lungs (17, 19), whereas cluster 3 *Col1a1*-expressing fibroblasts were enriched (20) (Supplemental Figure 2).

### PCs and Ig production in human lung grafts with BOS.

**Figure 3:**
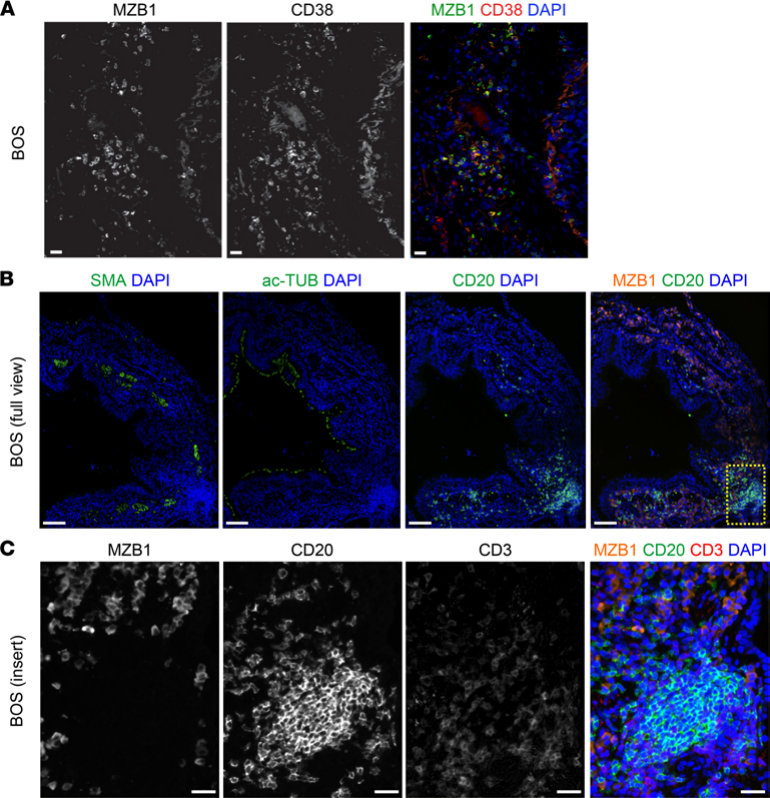
MZB1^+^ PCs are present around the airways in human BOS. (**A**) Immunofluorescence (IF) staining of MZB1 and CD38 in lung tissue samples with BOS after LTx (samples from the University of Colorado collection). Scale bar: 25 μm (**B** and **C**) IF staining of MZB1^+^ cells and their location relative to CD20^+^ and CD3^+^ cells around the remodeled airways (marked by the expression of α-smooth muscle actin and acetylated tubulin [ac-TUB]) in lung tissue samples with BOS after LTx (samples from the University of Colorado collection). (**B**) Full view; Scale bars: 250 μm. (**C**) Insert; Scale bar: 25 μm.

**Figure 4:**
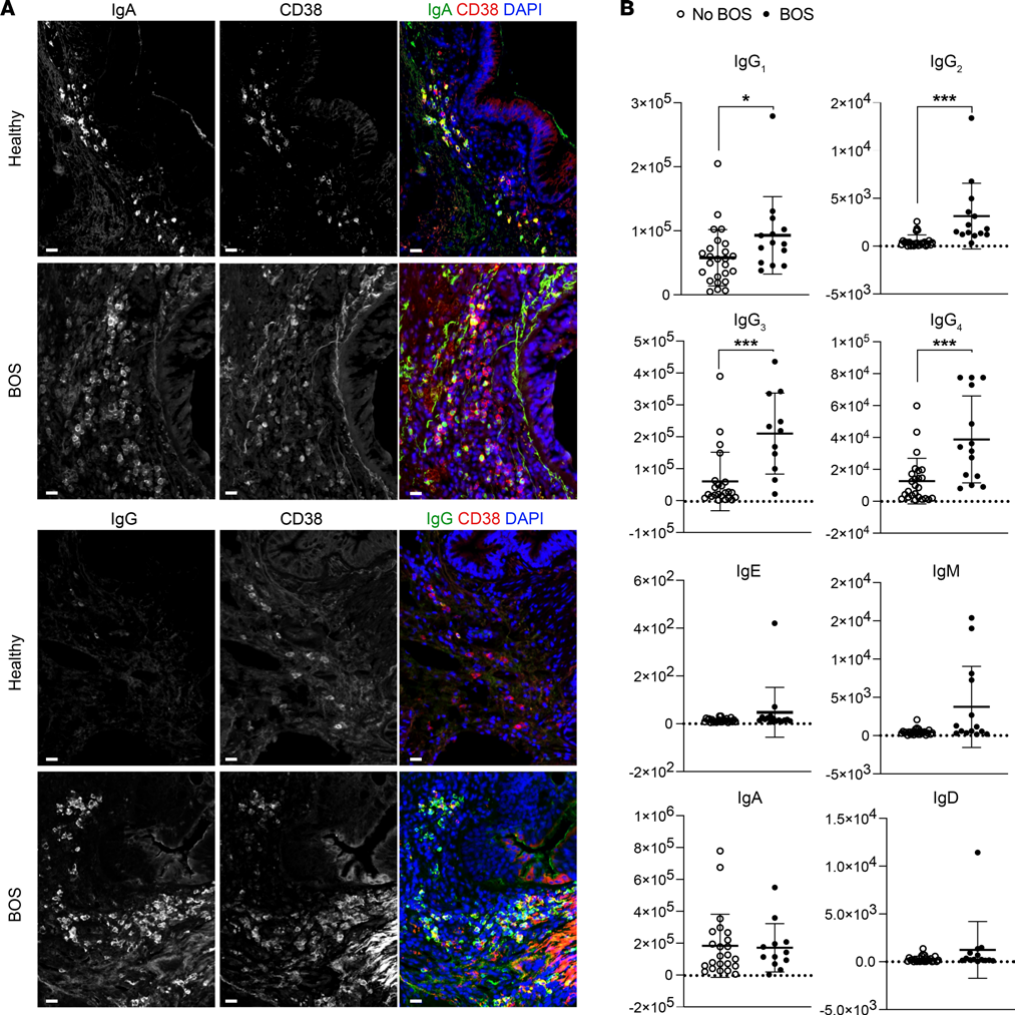
IgG^+^ PCs and local IgG are signatures of human BOS. (**A**) Immunofluorescence staining of IgA and CD38 in healthy donor and BOS tissue samples and staining of IgG and CD38 in healthy and BOS tissue samples (samples from the University of Colorado collection). Scale bars, 25 μm. (**B**) Quantification of the local Ig profile in BALF samples from patients with and without BOS after LTx (University of South Florida/Tampa General Hospital LTx cohort). Data are presented as mean ± SEM and analyzed with a Mann-Whitney unpaired, 2-tailed test. *P < 0.05; ***P < 0.001.

To assess the clinical relevance of the increased PC population in BOS, we compared explants from patients who developed BOS grade 3 and underwent retransplantation, with healthy control lungs (*n* = 10 each). PCs, identified by the expression of MZB1, CD38, CD138, and CD27, were robustly detected in large amounts in the peribronchial areas in BOS (Figure 2D, Figure 3A, and Supplemental Figure 3). Of note, these PCs were CD20^–^ and were found in large numbers in areas that were low in CD20 numbers (Figure 3, B and C). Although these peribronchial PCs displayed similar IgA expression in healthy and BOS samples, numerous IgG^+^ PCs were observed around the airways in BOS but were undetectable in healthy samples (Figure 4A). Quantification of local Ig isotype production in the bronchoalveolar lavage fluid (BALF) samples from patients with or without BOS who had undergone LTx (Table 1) showed that the titers of all 4 IgG subclasses (IgG_1_, IgG_2_, IgG_3_, IgG_4_) were significantly increased in BOS, whereas no differences were detected for IgE, IgM, IgA, or IgD isotypes comparing patients with and without BOS who had undergone LTx (Figure 4B).

### Heterogeneity of B cells in lung grafts.

**Figure 5:**
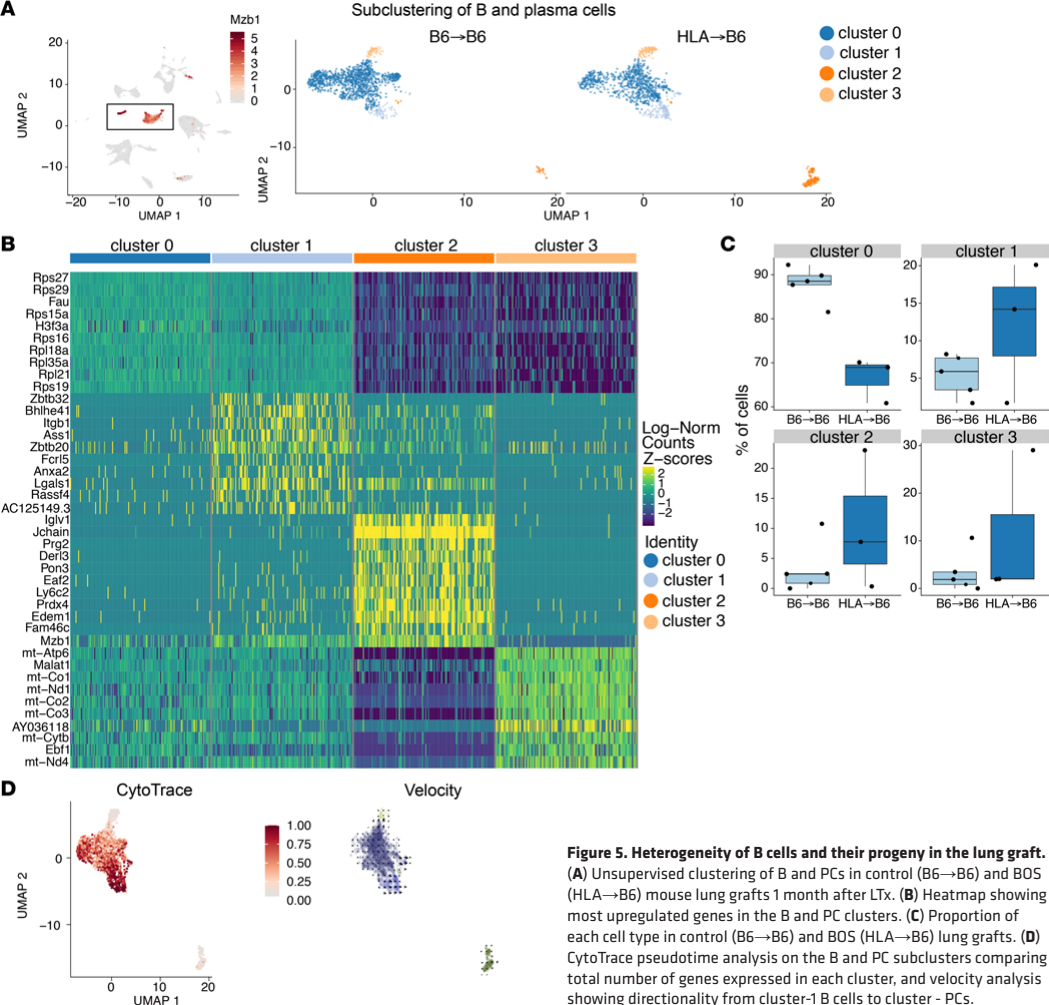
Heterogeneity of B cells and their progeny in the lung graft. (**A**) Unsupervised clustering of B and PCs in control (B6→B6) and BOS (HLA→B6) mouse lung grafts 1 month after LTx. (**B**) Heatmap showing most upregulated genes in the B and PC clusters. (**C**) Proportion of each cell type in control (B6→B6) and BOS (HLA→B6) lung grafts. (**D**) CytoTrace pseudotime analysis on the B and PC subclusters comparing total number of genes expressed in each cluster, and velocity analysis showing directionality from cluster-1 B cells to cluster - PCs.

An unsupervised detailed analysis of B and PC populations in mouse lung grafts identified 4 distinct subpopulations: 3 B cell clusters (clusters 0, 1, and 3) and a PC cluster (cluster 2) (Figure 5A). The majority of cells were found in cluster 0, and their numbers were similar between controls and BOS (n = 226 and 254 cells, respectively, in B6→B6 and HLA→B6 lung grafts). Cell numbers in clusters 1 and 3 were increased 4- and 3-fold in HLA→B6 versus B6→B6, respectively. Cluster 2, identified as the PC cluster, based on the expression of *Xbp1*, *Mzb1*, *Sdc1*, and *Prdm1*, displayed a 10-fold increase in cell numbers in HLA→B6 versus B6→B6 lung grafts (Figure 5, B and C). Pseudotime and velocity analyses were used to interrogate the origin of these PCs in the HLA→B6 lungs (Figure 5D). Pseudotime analysis, using CytoTrace (21), which identifies stem and differentiated cells on the basis of the total numbers of genes expressed, showed that PCs express fewer genes, whereas cluster 1 B cells expressed the most genes compared with the general population. This suggests that cluster-2 PCs are in a highly differentiated state, whereas cluster-1 cells are likely a progenitor population. The velocity analysis showed a directionality between cluster 1 and cluster 2 PCs, suggesting that PCs originate from cluster-1 B cells. The gene *Bhlhe41* was 1 of the predominant markers of cluster-1 cells. We validated, by immunostaining, the infiltration of BHLHE41^+^ cells around the airways of both humans and mice who developed BOS after LTx (Supplemental Figure 4).

### Functional characterization of the cluster-1 B cell subset.

To better understand the origin and functions of PCs in BOS, we investigated the cells from cluster 1, identified as potential progenitors of PCs in the BOS lung graft. Cluster-1 B cells expressed a number of transcription factors: *Zbtb32*, described in memory B cells, but absent in PCs; *Zbtb20*, expressed in B-1 cells and peaking in PCs (22); and *Bhlhe41*, recently unraveled as a marker of the B-1 population, which regulates B-1 cell differentiation, homeostasis, and function (23).

**Figure 6:**
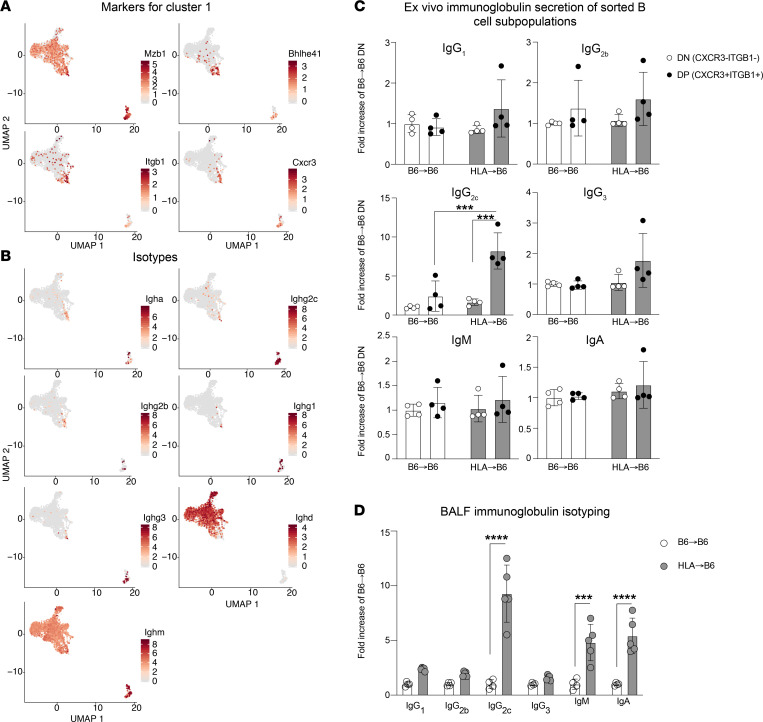
Functional characterization of the cluster-1 B cell subset. (**A**) Gene expression patterns projected onto UMAP plots of *Mzb1*, *Bhlhe41*, *Itgb1*, and *Cxcr3* in the B and PC subclusters from control (B6→B6) and BOS (HLA→B6) lung grafts. (**B**) Gene expression patterns projected onto UMAP plots of Ig isotypes in the **B** and PC subclusters from control (B6→B6) and BOS (HLA→B6) lung grafts. (**C**) Quantification of Ig isotypes in the conditioned media from CD19^+^CXCR3^+^ITGBB1^+^ and CD19^–^CXCR3^–^ITGB1^–^ cells sorted from HLA→B6 and B6→B6 mouse lung grafts 1 month after LTx, stimulated by LPS (1 μg/mL). Data are presented as mean ± SEM and analyzed with a 2-way ANOVA test followed by a Tukey’s multiple comparisons test. (**D**) Quantification of Ig isotypes in the BALF samples collected from the lung grafts of HLA→B6 and B6→B6 mice 1 month after LTx. Data are presented as mean ± SEM and analyzed with a 2-way ANOVA test followed by a Tukey’s multiple comparisons test. ***P < 0.001, ****P < 0.0001. DN, double negative CXCR3^–^ITGB1^–^; DP, CXCR3^+^ITGB1^+^.

On the basis of their first 50 marker genes, cluster-1 B cells were defined as an innate B-1 cell population, according to the Immunological Genome Project (ImmGen) database (24). In addition, cluster 1 was characterized by the expression of the surface markers *Cxcr3* and *Itgb1*, involved in lymphocyte homing and adhesion, respectively, and without any previously reported functional role in B-1 cells (Figure 6A). The expression of *Cxcr3* and *Itgb1* was significantly higher in cluster 1 than in the other clusters, with a 6- and 7-fold higher expression, respectively, compared with the other clusters. We used these surface markers, combined with the B cell marker CD19 in flow cytometry, to distinguish cluster 1 cells (CD19^+^CXCR3^+^ITGB1^+^) from the other clusters (CD19^+^CXCR3^–^ITGB1^–^). At 1 month after LTx, HLA→B6 lung grafts presented an increase in the number of CXCR3^+^ITGB1^+^IgG^+^ B cells (Supplemental Figure 5A). All Ig isotypes, with the exception of IgD, were expressed in cluster 1 and PCs at the gene level (Figure 6B). The absence of expression of IgD in cluster-1 cells is consistent with a B-1 phenotype.

To determine if cluster-1 B cells were capable of secreting Ig, we sorted these cells from mouse lungs with or without BOS 1 month after LTx and cultured them in the absence or presence of LPS (1 μg/mL) stimulation. A phenotypic analysis by flow cytometry confirmed that cluster-1 cells express the B-1a cell markers CD43 and CD5 (Supplemental Figure 5B). Cluster 1 cells secreted significantly higher amounts of IgG_2c_ compared with the other B cells, and this secretion was increased when the cells were sorted from lungs with BOS compared with controls (Figure 6C). The secretion of IgG_2c_ was also significantly increased in the BALF collected from lung grafts from HLA→B6 versus B6→B6 mice (Figure 6D). Altogether, we thus identified a B cell subpopulation with B-1 markers that infiltrates rejected lung grafts and is accountable for an increased local production of IgG_2c_.

### Causal role of non-IgM Abs in the development of BOS in vivo.

The observations of increased local Ab production in humans and mice with BOS prompted us to investigate their functional contribution to remodeling in chronically rejected lungs, which is still subject to debate. To address this, we transplanted HLA lungs to *Aicd^+/–^* or *Aicda^–/–^* mice (25), which are genetically deficient for the AID recombinase and production of all Igs except IgM. After clamping the right bronchus, the specific lung function of the left lungs was measured 1 month after LTx; naive, nontransplanted *Aicda^+/–^* littermates served as controls.

**Figure 7:**
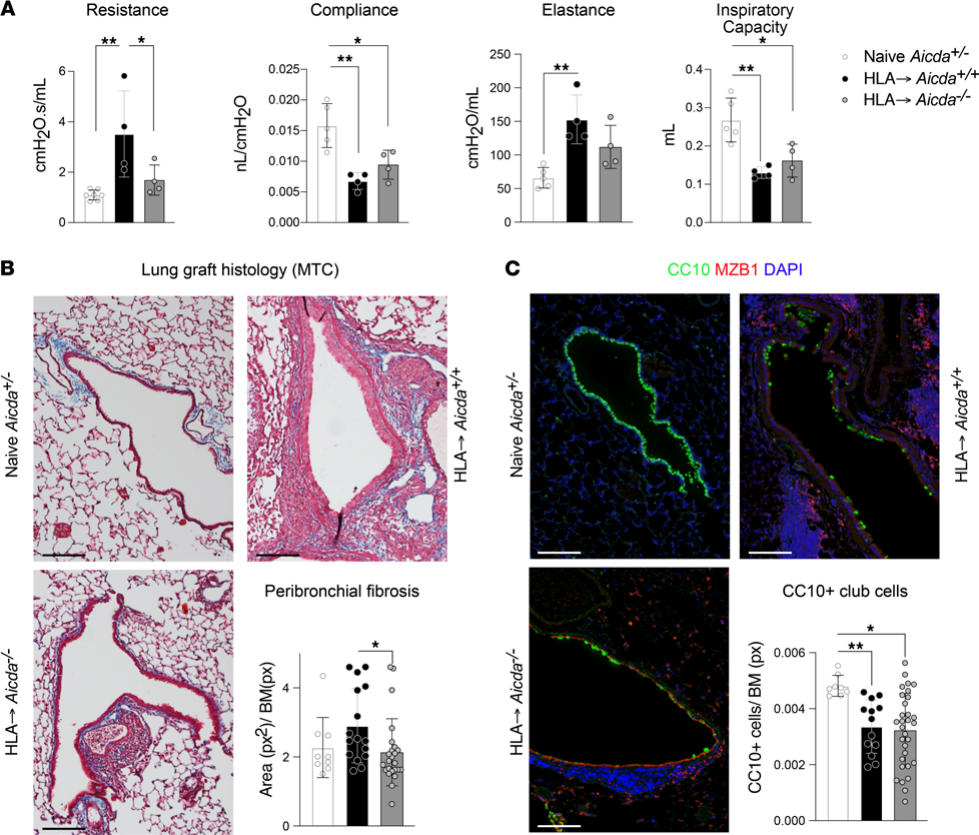
Genetic deficiency in Ab production partially protects murine lungs against the development of BOS signs. Lung grafts from HLA donors were orthotopically transplanted into *Aicda^+/+^* and *Aicda^–/–^* littermates on a B6 background (HLA→*Aicda^+/+^* and HLA→*Aicda^–/–^*) and analyzed 1 month later. Naive nontransplanted *Aicda^+/–^* mice were used as baseline controls. (**A**) Measurement of the lung function of the left lung grafts after clamping the right bronchus. (**B**) Representative histology after Masson trichrome (MTC) staining and quantification of the peribronchial fibrosis area, normalized to the basement membrane (BM) length. Scale bars, 100 μm. (**C**) Immunofluorescence staining for MZB1^+^ and CC10^+^ (club cell 10 kDa protein-positive) cells, and quantification of CC10^+^ cells normalized to the basement membrane length. Scale bars, 100 μm. Data are expressed as mean ± SEM and analyzed with a 1-way ANOVA followed by a Tukey’s multiple comparisons test. *P < 0.05, **P < 0.01. px, pixel.

Lung function was significantly improved when HLA lungs were transplanted into *Aicda^–/–^* mice compared with HLA→*Aicda^+/+^* mice (Figure 7A). In particular, pulmonary resistance was significantly lower in HLA→*Aicda^–/–^* grafts compared with HLA→*Aicda^+/+^* (Figure 7A). In addition, peribronchial fibrosis was significantly attenuated in HLA→*Aicda^–/–^* grafts compared with HLA→*Aicda^+/+^* (Figure 7B). The numbers of CC10^+^ cells remained unchanged between HLA→*Aicda^+/+^* and HLA→*Aicda^–/–^* grafts (Figure 7C). Altogether, our results demonstrate that the prevention of Ab production (with the exception of IgM) has a beneficial impact on lung graft function after LTx, potentially through decreased fibrogenesis around the airways.

## Discussion

We applied single-cell analysis with unsupervised clustering to identify cell-subset composition in murine lung grafts during the progression of chronic rejection after orthotopic LTx. This analysis generated 14 large cell clusters identified as 11 known cell types. Subclustering of lung structural cells resulted in 11 additional clusters. We identified cell types previously detected in the mouse lung by the scRNA-Seq technique (26) and observed dramatic shifts in the relative abundance of both immune and structural cell types between chronically rejected and control transplanted lungs. The gene expression signatures of these clusters offer molecular determinants in the progression of chronic rejection to BOS after LTx.

To induce chronic rejection after LTx, we used a previously published mouse model, based on orthotopic LTx of donor grafts from mice that contain a knock-in of the human HLA-A2 to C57BL/6J recipients. This model does not induce (hyper)acute rejection, typically encountered in the context of xenotransplantation. Instead, the human HLA-A2 in the donor organ triggers an indirect allorecognition response in C57BL/6J recipients (27), also seen in BOS (28, 29). We previously reported that HLA→B6 lung grafts share common hallmarks, and lung function changes with human BOS (i.e., LB, deterioration of graft lung function, partial loss of CC10^+^ cells, and peribronchial fibrosis in this model) (4, 30).

During BOS, PCs, identified by the expression of the classical PC markers *Xbp1*, *Sdc1*, *Mzb1*, and *Irf4* (31), are the cell type exhibiting the most prominent numeric increase compared with control mouse lung grafts. Accumulations of MZB1^+^ PCs were consistently detected around the airways of explanted lung samples in patients with BOS and mice after LTx. Although we occasionally detected PCs in lung tissue explanted from healthy donors, these were much less abundant and only positive for IgA, whereas PCs in BOS samples were mostly IgG^+^. PCs represent the terminal stage of B cell differentiation and are known for their ability to produce large amounts of Abs (32). We showed that total IgG levels are increased in BALF of patients with BOS after LTx, compared with patients who remained BOS-free. This finding, combined with the presence of IgG^+^ PCs around BOS airways, suggests a local origin of the soluble IgG measured in BALF, highlighting that a local Ab repertoire drives transplant rejection (33, 34). This significant increase of overall IgG levels in association with BOS is consistent with previously published data (35) yet extends those results to increased detection of individual Ig isotypes (IgG_1_, IgG_2_, IgG_3_, and IgG_4_). Increasing evidence shows that the local (intragraft) and systemic (peripheral blood) Ab repertoires are uncoupled in terms of specificity, abundance, and isotype predominance. The presence of donor-specific Abs (DSAs) inside the graft differs from that in peripheral blood in chronic lung allograft dysfunction (CLAD) (33), aligning with our finding that absence of systemic DSAs does not infer that BALF IgGs are not DSAs. Accumulating data highlight an autoreactive or polyreactive repertoire of intragraft Ig, as documented in cardiac allograft vasculopathy (36) or Ab-mediated rejection after kidney transplantation (37). The assessment of BALF Ig specificity and its overlap with the B cell receptor repertoire of MZB1^+^ cells will thus constitute a focus area for future investigations.

Although an association between Ab levels (peripheral and local) and BOS has been reported by us and others, their functional contribution to disease onset and progression remains unknown. To mechanistically address this question, we performed orthotopic LTx in *Aicda^–/–^* mice, which lack secretion of all Ab types except IgM (which was unchanged during chronic rejection). We determined that the absence of those Ab subclasses in *Aicda^–/–^*-recipient mice was sufficient to significantly inhibit peribronchial fibrosis and lung function changes associated with increased graft airway resistance after LTx. The absence of class-switched Abs did not affect the loss of club cells evident in our model and characteristic for BOS. Our results support the crucial impact of Abs in the pathogenesis of CLAD, particularly in light of previous findings showing that lack of Abs, including IgM, is protective in a mouse model of CLAD. In addition to the prevention of fibrosis, the Ab-deficient mice were also protected from club cell loss in a mouse model of CLAD (38). Studies are needed to determine whether Ab functions in this context are Fc dependent (complement-mediated cytotoxicity, FcγR-dependent inflammation) (39) or Fab dependent (direct activation of target cells through endogenous surface proteins, which can be either MHC molecules or surface autoantigens) (40–43).

The PCs detected in the lungs during BOS development were numerous around fibrotic and partially obliterated airways. Hence, we sought to identify the potential cellular origin of these peribronchial PCs by subclustering B cells and PCs identified by scRNA-Seq of mouse lung grafts (Figure 3). We identified the likely cellular origin of PCs in rejected lung grafts using trajectory and pseudotime analysis. These PC progenitors were defined by a set of transcripts encoding the cell surface markers integrin β 1 (ITGB1) and CXCR3 [known to regulate cell adhesion (44) and chemotaxis (45, 46)], as well as the transcription factors BHLHE41, ZBTB20, and ZBTB32. In murine lung grafts, both the PC cluster, as well as the cells’ putative progenitors, were the only intragraft B cells positive for genes encoding IgA, IgG_1_, IgG_2b_, IgG_2c_, or IgG_3_. In BALF, IgA and IgG_2c_ levels were significantly increased compared with controls. IgA, mostly produced around the bronchial epithelium in steady state, is considered protective, because it is the first line of defense against airborne pathogens (47). IgA, therefore, is unlikely to contribute to rejection after LTx, although in some rare cases, IgA has been found capable of contributing to autoimmunity, as in the case of IgA nephropathy (48). We sorted CXCR3^+^ITGB1^+^ PC progenitors from mouse lung grafts and demonstrated that this population specifically contributed to the increased production of IgG_2c_. The IgG_2c_ is mainly generated in response to carbohydrates and proteins (39) and provides protection against bacteria and viruses (49, 50), but its contribution to chronic rejection was hitherto unknown.

Using the ImmGen database (24), we determined that PC progenitors identified in rejected mouse lung grafts display a B-1 phenotype. This is based on their expression of the classical B-1 markers *Cd43* and *Ighm,* as well as *Bhlhe41*, a transcription factor recently identified as a marker of B-1 cells, shaping their development, homeostasis, and BCR repertoire (23). In addition to these conventional markers, this BOS-associated B-1 cell population displayed markers previously undefined or unknown in the characterization and/or function of B-1 cells, such as *Cxcr3* and *Itgb1*. The transcriptome of the chronic rejection–associated B-1 cell population did not overlap much with innate response activator B cells, another B-1 cell population, that was discovered to regulate several pathological contexts (Supplemental Figure 6) (51).

Therefore, our data identify what we believe to be a novel role of the B-1 cell population in BOS (52–54). B cells are classically subdivided in conventional B-2 cells and innate-like B-1 cells (55, 56). Conventional B-2 cells are the prototype of adaptive humoral immunity, selected for the production of Abs with the highest specificity and affinity against encountered antigens. Although the involvement of B-2 cells in infectious and chronic inflammatory diseases has been extensively investigated, the role of B-1 cells in disease has been underappreciated. Innate B-1 cells are distinguished from B-2 cells by their different developmental origin, a self-renewal capacity, in situ location, and a number of phenotypic and functional characteristics. Although the Ab repertoire of B-2 cells constantly evolves upon antigen encounters, the B-1 repertoire in mostly predetermined during prenatal development (57). B-1 cells produce polyreactive Abs (i.e., having low affinity and broad specificity) targeting common pathogenic motifs or intracellular autoantigens (57). B-1 cells secrete natural IgM, but recent evidence, including our findings, shows that they can also switch Ab classes and produce IgG (58). Although B-1 cells and their natural IgM have beneficial effects in, for example, atherosclerosis (59), the intrinsically autoreactive nature of their Ab repertoire also assigns to them a recently described role in autoimmunity (60), in particular due to the generation of auto-Abs in transplantation (60, 61). Autoimmunity is associated with BOS after LTx, as evidenced by increased production of anti-collagen V auto-Abs and their potential to induce BOS (62, 63). Our data suggest that a subset of innate B-1 cells, identified by classical markers (*Bhlhe41*, *Cd43*, and *IgM*) and context-specific markers (*Cxcr3* and *Itgb1*), can contribute to autoimmunity in BOS, and thus represent potential therapeutic value. Polyreactive Abs have recently been associated with cardiac allograft vasculopathy after heart transplantation (36, 64). More recently, innate B cells have been detected in rejected human kidney grafts (37). Of note, the phenotype of intrarenal innate B cells was independent of serum DSAs, and their profile was mostly autoreactive, which suggests a distinct role for this B cell population compared with DSA-producing B cells classically incriminated in allograft rejection. To our knowledge, our report is the first to date in which a set of unique markers is identified for this innate B cell population and that unravels a mechanistic contribution of this local, innate B cell subpopulation to chronic graft dysfunction and rejection. Importantly, in addition to the production of potentially pathogenic Abs, these subpopulations of B-1 cells can also produce protective IgM (65), regulate inflammatory processes (54) and T cell differentiation and function (56), or even directly interact with structural cells to influence their function in disease (66). In conclusion, our results identify in lung grafts undergoing chronic rejection after LTx a CXCR3^+^ITGB1^+^ B-1 cell subset that are progenitors to intragraft MZB1^+^ PCs capable of producing high levels of IgG_2c_ in association with the development of BOS.

## Methods

### Patient demographics

#### University of South Florida/Tampa General Hospital LTx cohort.

Samples of BALF from patients who developed BOS or who did not develop BOS after LTx, provided by Nirmal Sharma (University of South Florida/Tampa General Hospital, and Brigham and Women’s Hospital, Harvard Medical School, Boston, MA, USA) and Kapil Patel (University of South Florida/Tampa General Hospital), were from a sample of the Tampa General Hospital LTx cohort. The demographics are presented in Table 1.

#### University of Colorado collection.

Paraffin-embedded samples of human BOS lung tissue were provided in-house. The tissue was explanted from 4 female and 6 male patients who underwent a LTx and received a second transplant at the UCHealth University of Colorado Hospital (Aurora, CO, USA) after they developed end-stage BOS. Paraffin-embedded samples of healthy lung tissue collected from organ donors served as controls.

### Orthotopic LTx in mice

Male C57BL/6 (B6) and HLA (*C57BL/6-Tg(HLA-A2.1)^1Enge/J^*) mice were purchased from The Jackson Laboratory. *Aicda^–/–^* (*Aicda^tm1Hon^*) mice were a gift from Dr. Jing Wang (University of Colorado, Anschutz Medical Campus, Aurora, CO, USA). All mice were backcrossed on a C57BL/6J background for at least 10 generations. LTxs were performed as described previously (4) with minor modifications. No immunosuppression was applied to any of the transplanted mice. B6 and HLA mice were used as donors, and B6 mice were used as recipients. Briefly, donors were anesthetized with an i.p. injection of ketamine and xylazine. The pulmonary artery, bronchus, and pulmonary vein were carefully separated from one another with blunted forceps, prior to cuffing with, respectively, 24, 20, and 22 gauge cuffs. The left lung graft was stored for less than 1 hour before its implantation. The recipient mouse was anesthetized with a mixture of medetomidine (1 mg/kg), midazolam (0.05 mg/kg), and fentanyl (0.02 mg/kg); intubated; and connected to a small-animal ventilator (Harvard Apparatus) at a respiratory rate of 120 bpm and a tidal volume of 300 μL. The chest was opened on the left side between ribs 3 and 4, and the native left lung was retracted with a clamp. The hilar structures were carefully separated from one another with blunted forceps. After arrest of the blood and air flow toward the left lung, the cuffed graft pulmonary artery, bronchus, and pulmonary vein were inserted into the recipient counterparts and ligated with 9-0 sutures. The native left lung was removed, and the incision in the chest was closed with a 6-0 suture after removing all potential air bubbles from the chest. Antagonist was administrated, and the animal was extubated when it showed signs of spontaneous breathing. After the operation, the recipient mice were allowed to recover at 30°C overnight and received a single injection of buprenorphine SR (1 mg/kg). The mice were sacrificed at 1-month after LTx.

### scRNA-Seq analysis of murine lung grafts

#### Preparation of murine lung grafts as single-cell suspensions.

Single-cell suspensions of murine lung grafts were used for the scRNA-Seq analysis, flow cytometry, and FACS. The graft lung tissue was digested and separated into single-cell suspensions using the Lung Dissociation Kit, Mouse (Miltenyi), according to the manufacturer’s instructions.

#### scRNA-Seq.

Single cells were captured using the 10x genomics chromium platform. A total of 10,000 cells were loaded and single-cell libraries were generated using the 10x genomics 3′-end gene expression kit. Libraries were sequenced to a depth of 650 million to 1 billion reads, resulting in 94.2% to 97.9% sequencing saturation.

#### scRNA-Seq analysis.

Sequencing data were processed using the Cell Ranger pipeline from 10x Genomics with the mm10 genome assembly to generate unique molecular identifier (UMI) count matrices for each sample. The *Seurat* package was used to perform normalization, clustering, and to generate UMAP projections (67). Cell barcodes containing fewer than 200 UMIs and genes detected in fewer than 3 cells were excluded. The R package *SoupX* was used to remove free RNA contamination (68), using *Hbb-bs*, *Hba-a1*, *Hba-a2*, *Hbb-bt*, *H2-Ab1*, *H2-Aa*, *H2-Ab1*, and *H2-Eb1* as markers of erythrocytes; *Scgb1a1* as a club cell marker; *Cd74*, *Ighg2c*, *Igkc*, *Ighm*, and *Ighg3* as markers of B cells; *Sftpc* as a marker of type II cells; *Apoe*, *Lyz2*, and *Psap* as markers of macrophages; and *Ly6c1* and *Ly6a* as markers of other lung structural cell populations. Principal component analysis was performed on *z* score transformed data using 30 dimensions for the full data set and 20 dimensions for the structural lung cells and B cell subsets. Graph-based clustering was performed using resolutions of 0.1, 0.3, and 0.1 for the full data set, structural cells, and B cell subsets, respectively. The R package *clustifyr* was used to aid in assigning cell types to clusters (69). The Tabula Muris droplet mouse scRNA-Seq data set was used to define a reference data set for calling cell types (12). Average log-normalized expression values were calculated for cell types defined in the cell_ontology_class category.

Genes differentially expressed in each cluster compared with other clusters in each tested comparison were determined with a Wilcox rank-sum test from the *Presto* R package (70). Heatmaps were generated using *ComplexHeatmap* or *Seurat* (71). Pseudotime values were calculated using the CytoTrace algorithm, using the UMI count matrix as input (21). RNA velocity vectors were computed and plotted using the velocyto *Python* package (72).

#### Data and code availability.

Sequencing data, UMI count matrices, and cell-level metadata are deposited in the NCBI’s Gene Expression Omnibus database (accession # GSE166386). Analysis scripts and an interactive University of California, Santa Cruz, cell browser instance are provided at a GitHub repository (https://github.com/rnabioco/LTx-rejection-map; commit ID 02944e1).

### Measurement of Igs in human BALF

Levels of Igs in human BALF samples were measured with the LEGENDplex Human Immunoglobulin Isotyping panel (BioLegend, #740714), according to the manufacturer’s instructions.

### Flow cytometry

Single-cell suspensions from murine lung grafts were counted, then incubated with the following Abs: anti-mouse CD19-PerCP Cy5.5 (eBioscience, Thermo Fisher, clone eBio1D3, #45-0193-80), anti-mouse CD183(CXCR3)-PE, mouse (Miltenyi Biotec, clone REA724, #130-111-087), anti-mouse CD29(ITGB1)-APC, mouse (Miltenyi Biotec, clone HMβ1-1, #130-102-557), BD Pharmingen PE anti-mouse CD43 (BD Biosciences, clone S7, #561857), and anti-mouse CD5-APC (eBioscience, Thermo Fisher, clone 53-7.3, #17-0051-81). Fluorescence was quantified using a FACS Canto II flow cytometer (BD Biosciences) and analyzed with the DIVA software (BD Biosciences).

### FACS and ex vivo stimulation of B cell subpopulations

Single-cell suspensions from murine lung grafts were counted, then incubated with the following Abs: anti-mouse CD19-PerCP Cy5.5 (eBioscience, Thermo Fisher, clone eBio1D3, #45-0193-80), anti-mouse CD183(CXCR3)-PE, mouse (Miltenyi Biotec, clone REA724, #130-111-087), anti-mouse CD29(ITGB1)-APC, mouse (Miltenyi Biotec, clone HMβ1-1, #130-102-557). The cells were sorted into cooled sterile PBS+10% FBS, into 2 cell populations—CD19^+^CXCR3^+^ITGB1^+^ and CD19^+^CXCR3^–^ITGB1^–^—using the BD Aria I cell sorter (BD Biosciences). The cells were centrifuged at 300*g*, then resuspended in RPMI, supplemented with 10% FBS, penicillin and streptomycin, 2 mM glutamine, 0.1 mM nonessential amino acids, 1 mM sodium pyruvate, and 50 μM 2-mercaptoethanol. The cells seeded in a round-bottom 96-well plate and cultured for 5 days with LPS (1 μg/mL) at 37°C in 5% CO_2_. The supernatants were collected for Ig measurement.

### Measurement of murine Igs

Igs were measured in preconditioned media from LPS-stimulated B cell subpopulations sorted from murine lung grafts. The levels of IgG_2c_ were measured using the IgG_2c_ Mouse ELISA kit (Thermo Fisher Scientific, #88-50670-22), according to the manufacturer’s instructions. The levels of IgG_1_, IgG_2b_, IgG_3_, IgA, IgM, and IgD were measured using the Mouse Immunoglobulin Isotyping kit (BD, #550487) according to the manufacturer’s instructions.

### Histology and immunostaining

Sections of paraffin-embedded specimens were acquired from the Pathology core of the University of Colorado. Mouse lung grafts were fixed with PFA 4% instilled intratracheally for 24 hours. The specimens were embedded in paraffin and prepared as 4 um thick sections at the Histology Core of the National Jewish Hospital, Denver, CO, USA. Both human and mouse paraffin sections were deparaffinized by melting the paraffin at 60°C, followed by incubations in sequential xylene, ethanol, and water baths. Antigen retrieval was performed in 0.01 M citric acid, pH 6.0, in a pressure cooker for 30 seconds at 125°C. For IHC staining of MZB1, endogenous peroxidases were inhibited with 3% H_2_O_2_, then the Avidin/Biotin blocking kit (Vector Laboratories) was used. Sections were blocked with PBS+3%BSA, then incubated with the primary anti-MZB1 Ab (polyclonal, Sigma-Aldrich, #HPA043745-100UL) overnight at 4°C. Sections were incubated with a secondary biotinylated anti-rabbit Ab (Agilent Technologies), followed by peroxidase (ABC Vectastain kit, Vector Laboratories). MZB1 was finally detected with the DAB substrate (Vector Laboratories). The image acquisition was performed with the Aperio slide scanner (Leica Biosystems), and the eSlide manager software.

For immunofluorescence staining, sections were blocked with PBS+3%BSA, then incubated overnight at 4°C with the following primary Abs: anti-MZB1 (Sigma-Aldrich, #HPA043745-100UL), anti-CD38 (Santa Cruz Biotechnology, clone H-11, #sc-374650), anti-CD20 (Thermo Fisher, #14-0202-82), anti-CD3 (Bio-Rad, #MCA1477), α-smooth muscle actin (Santa Cruz Biotechnology, #sc-53142), anti–acetylated tubulin (Abcam, #ab24610), anti–human IgG (Abcam, clone EPR4421, #ab109489), and anti-IgA (Abcam, clone EPR-5367-76, #ab124716). Finally, the sections were incubated with fluorochrome-conjugated secondary Abs (Invitrogen: Anti-Rabbit Alexa Fluor 488, #A21206; Anti-Rat Alexa Fluor 488, #A11006; Anti-Mouse Alexa Fluor 488, #A21131; Anti-Rat Alexa Fluor 555, #A21434; Anti-Rabbit Alexa Fluor 568, A11036; and Anti-Mouse Alexa Fluor 568, A21134). The images were acquired with an Olympus microscope (BX43).

### Lung function measurements of left lung grafts

Mice were anesthetized with pentobarbital, then intubated and connected to a Minivent-ventilation system (120 breaths/min, volume, 300 μL). The chest was opened, and the right bronchus was clamped. Then, the left lung function was measured with a FlexiVent system (Scireq) (tidal volume, 3.75 mL/kg; frequency of 150 breaths/min). Airway compliance, resistance, tissue elastance, and inspiratory capacity were measured using the SnapShot, Prime-8, and Quick-prime wave perturbations. Three readings per animal were taken.

### Statistics

Data represent mean ± SEM, from *n* separate experiments. Statistical significance of differences was evaluated by a Mann-Whitney unpaired 2-tailed test or a 1-way ANOVA followed by a Tukey’s multiple comparisons posttest or a 2-way ANOVA followed by a Tukey’s multiple comparisons posttest. Differences were considered statistically significant at *P* < 0.05. The types of analysis used for each data set are indicated in the legends of the corresponding figures.

### Study approval

The use of the University of South Florida/Tampa General Hospital LTx cohort was approved by an IRB (IRB no. Pro00032158, University of South Florida).

For the University of Colorado collection, the use of patient samples with end-stage BOS was approved by a local IRB, Colorado Multiple Institutional Review Board (COMIRB; IRB# 18-0178). The use of healthy lung tissue collected from dead donors at the National Jewish Hospital was approved by COMIRB (IRB no. 11-1664).

All animal experimentations were approved by the IACUC of the University of Colorado [protocol 115517(04)2D].

## Author contributions

NFS designed the research studies, conducted experiments, acquired and analyzed data, and contributed to writing the manuscript. KR analyzed the scRNA-Seq data and contributed to writing the manuscript. MB designed research studies, acquired and analyzed data, and contributed to writing the manuscript. SC, PS, and XW acquired and analyzed data. KNP provided University of South Florida/Tampa General Hospital (USF-TGH) LTx cohort samples. CC provided the University of Colorado collection samples. MK contributed to writing the manuscript. NSS provided USF-TGH LTx cohort samples and edited the manuscript. OE designed research studies, analyzed data, and contributed to writing the manuscript.

## Supplementary Material

Supplemental data

## Figures and Tables

**Table 1:**
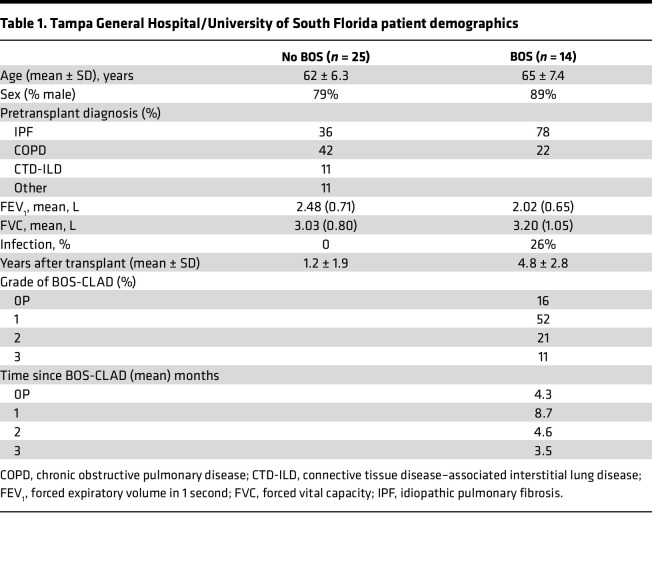
Tampa General Hospital/University of South Florida patient demographics

## Version 1 09/22/2022

Electronic publication
