# Supplementary material for: Single-cell transcriptome mapping identifies a local, innate B cell population driving chronic rejection after lung transplantation
Source: JCI Insight. 2022 Sep 22;7(18):e156648. doi: 10.1172/jci.insight.156648 (PMC9675462; doi:10.1172/jci.insight.156648)
Supplement: Supplemental data [file jciinsight-7-156648-s199.pdf]

Supplementary Material

- **Supplementary figure legends**
- **Supplementary figures**

Sup. Fig. 1. Annotation of cell populations from mouse lung grafts. A, Number of cells included in the single cell RNA-sequencing analysis per population and per sample. **B,** Heatmap for cell annotations of the different clusters after correlation to the gene expression profiles of the Tabula Muris (11). **C,** Uniform Manifold Approximation and Projection (UMAP) representation of 11 distinct cell populations from unsupervised clustering of gene expression data in single cells extracted from control (B6→B6) and BOS (HLA→B6) transplanted mouse lungs. Selected marker gene expression is represented according to the provided color scheme. **D,** Violin plots of log-transformed gene expression of selected cell population marker genes displayed for the control (B6→B6) and BOS (HLA→B6) transplanted mouse lung conditions.

Sup.Fig. 2. Analysis of structural lung cells from mouse lung grafts. The cell population identified as structural lung cells in the analysis of control (B6→B6) and BOS (HLA→B6) lung grafts was submitted to unsupervised subclustering. **A,** Uniform Manifold Approximation and Projection (UMAP) representation of 11 distinct cell populations from unsupervised clustering of gene expression data in single cells identified as structural lung cells extracted from control (B6→B6) and BOS (HLA→B6) transplanted mouse lungs (top). UMAP representation of the cell populations detected in murine lung grafts by condition (HLA→B6 and B6→B6) (bottom left). Proportion of each cell type in control vs. BOS mouse lung graft samples (bottom right). **B,** Violin plots of log-transformed gene expression of selected cell population marker genes

displayed for the control (B6→B6) and BOS (HLA→B6) transplanted mouse lung conditions.

Sup. Fig. 3. MZB1+ cells express classical plasma cell markers in human lung grafts with BOS. A, B, Immunofluorescence staining of MZB1, CD27 and CD138 in a sample with BOS.

Sup. Fig. 4. BHLHE41+ cells are a common feature of mouse and human lung grafts with BOS. A, Immunofluorescence staining of the hematopoietic marker CD45 and BHLHE41 in lung graft tissue from a BOS (HLA→B6) mouse. **B,** MZB1 and BHLHE41 in a lung tissue sample from a patient with BOS after LTx (samples from the University of Colorado collection). Scale bars are 50 um.

Sup. Fig. 5. Flow cytometry analysis of lung graft B cell populations. A, 1 month after lung transplantation (LTx), mouse lung grafts from control (B6→B6) and BOS (HLA→B6) were dissociated into single cells and the CD19+CXCR3-ITGB1-IgG+/- (non-cluster #1) and CD19+CXCR3+TGB1+IgG+/- (cluster #1) B cells were analyzed and sorted by FACS (top). **B,** The expression of CXCR3, ITGB1 and classical B-1 cell markers CD43, CD5 was confirmed after a 5 day-long ex vivo culture of the sorted B cell populations (bottom). Data are presented as mean +/- SEM. $*p<0.05$, Mann-Whitney t-test.

Sup. Fig. 6. Comparison of lung graft B cell subsets to available data. Data from the single cell RNA-sequencing analysis of B and plasma cell cluster identified in the mouse lung grafts was compared to the B cell subsets identified by Rauch and colleagues and deposited in the NCBI GEO database (series GSE32372), and presented as a correlation.

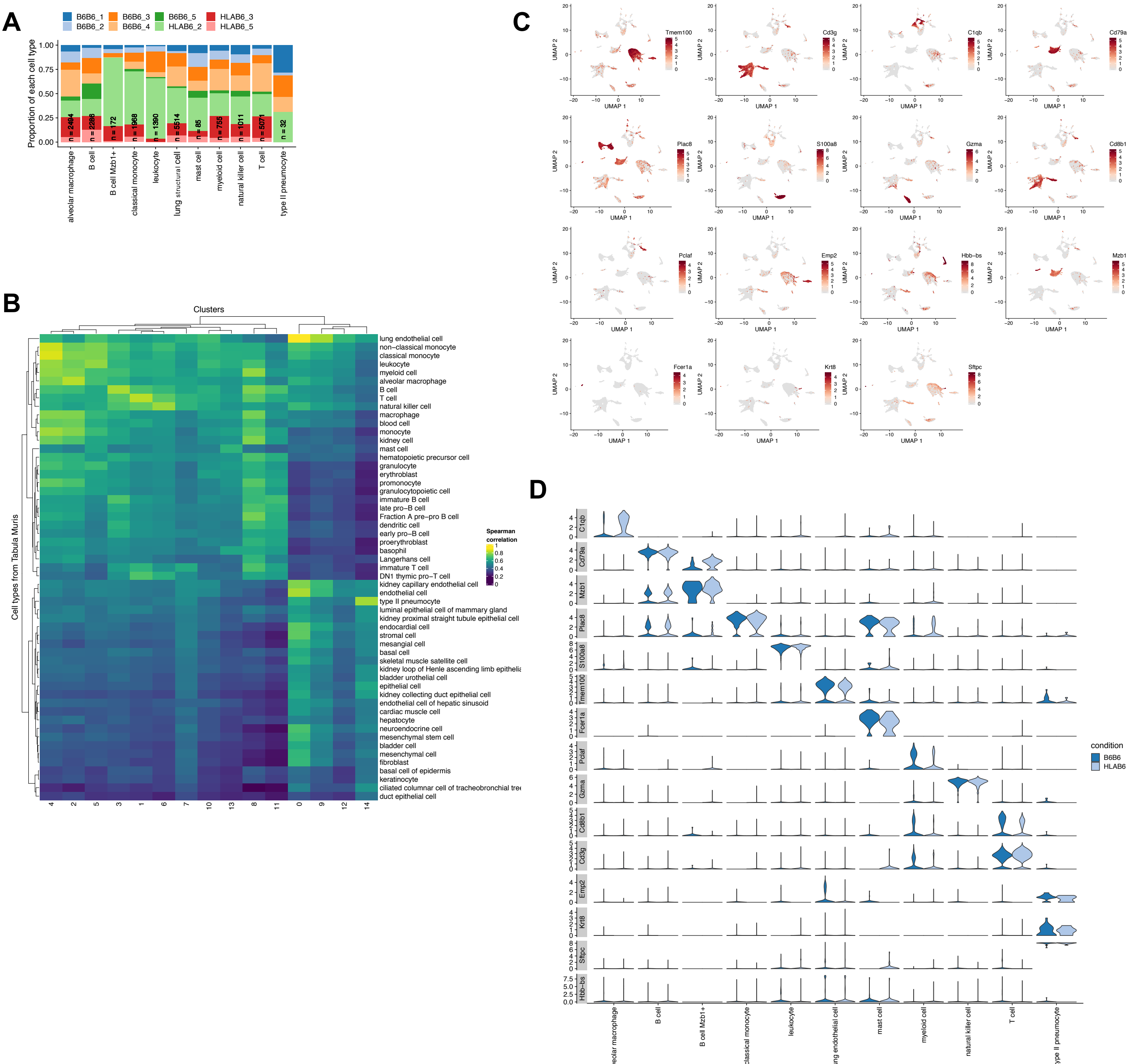

Supplementary Figure 1

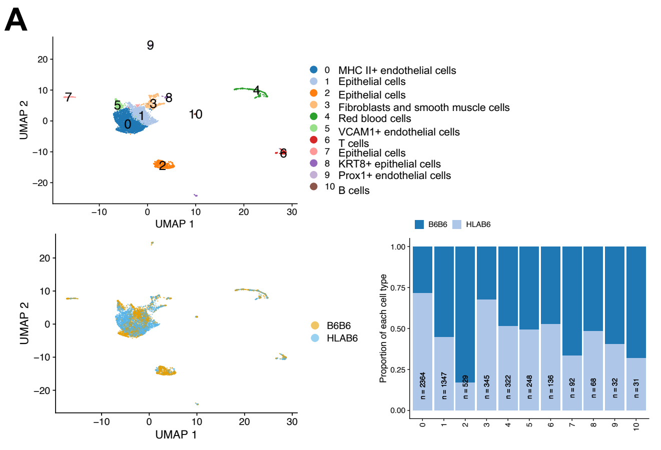

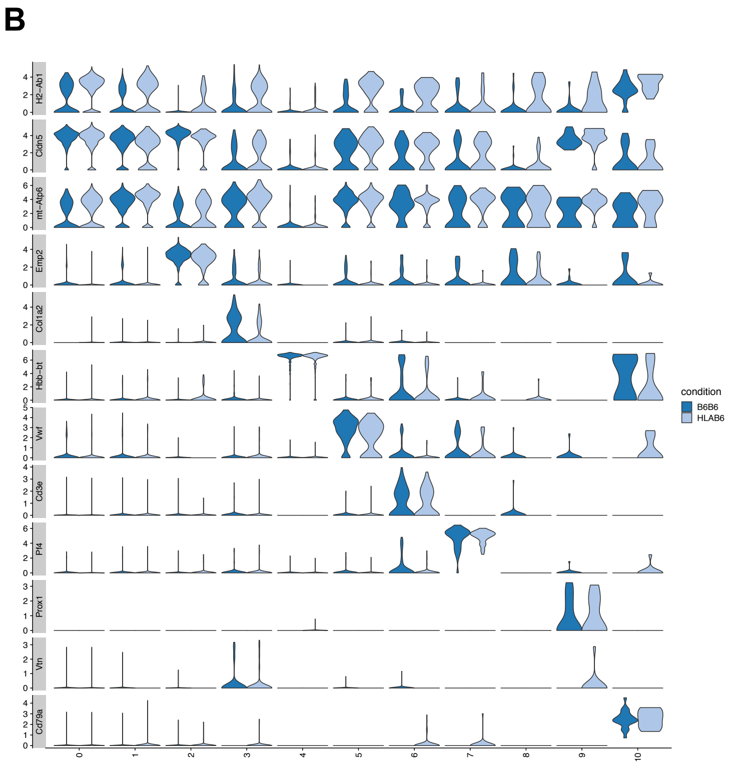

Supplementary Figure 2

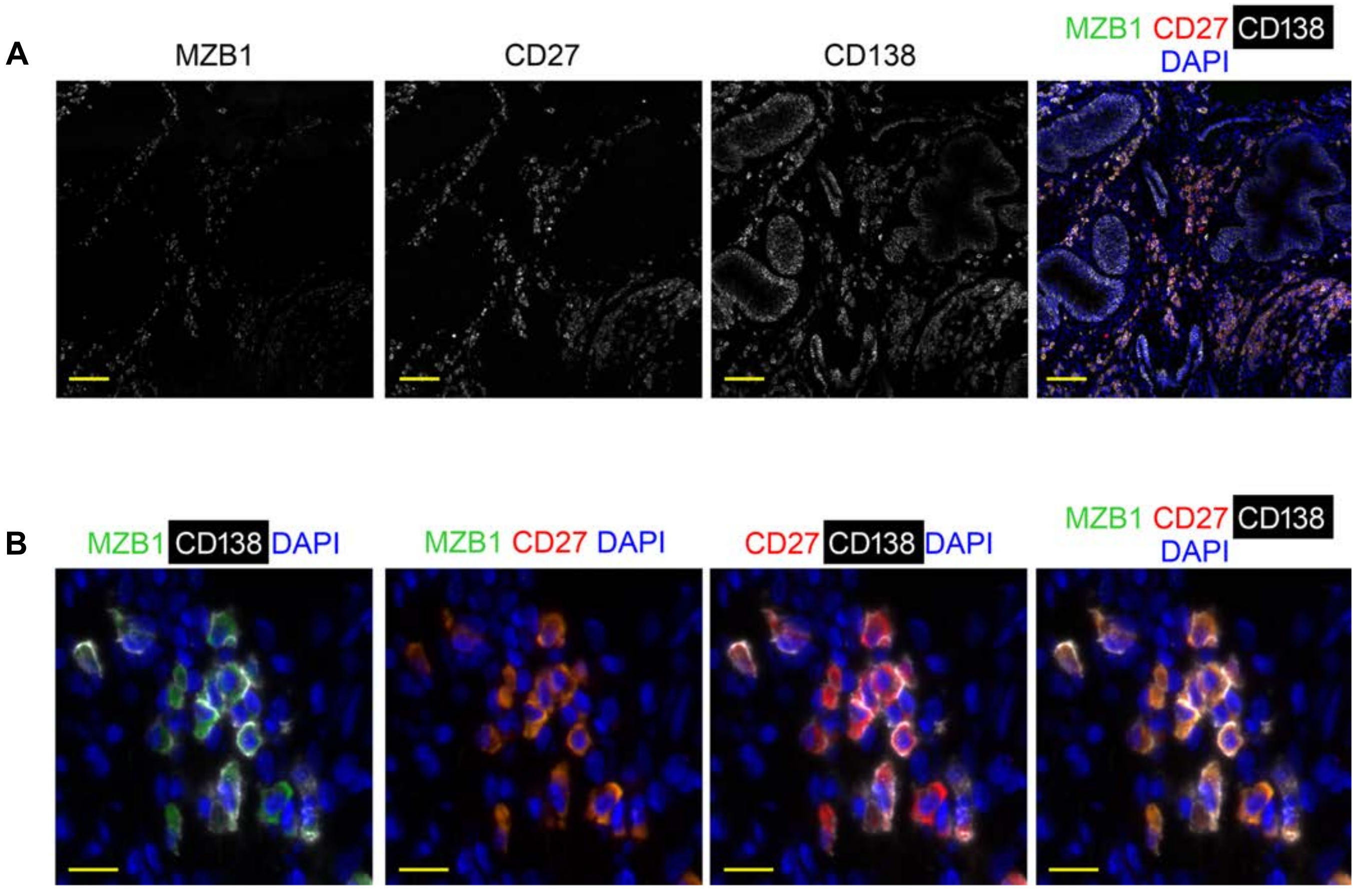

Supplementary Figure 3

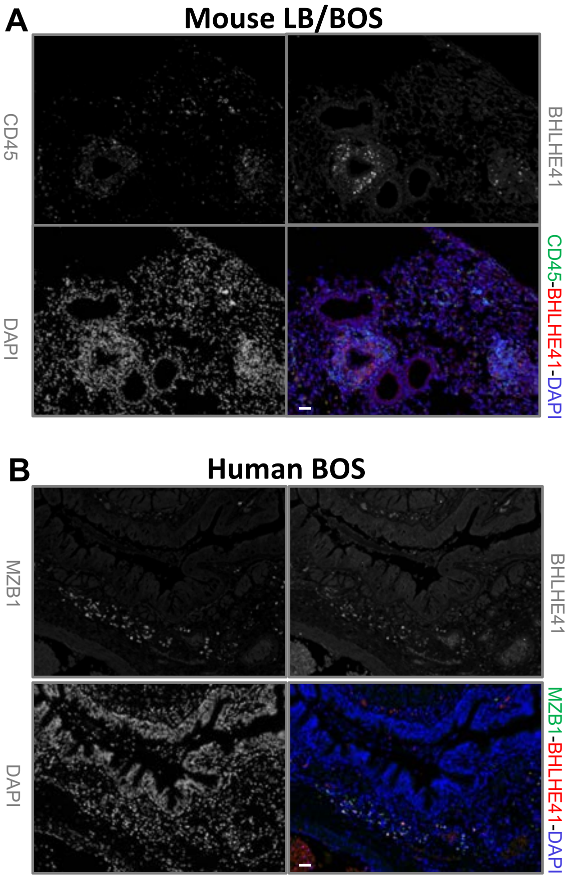

Supplementary Figure 4

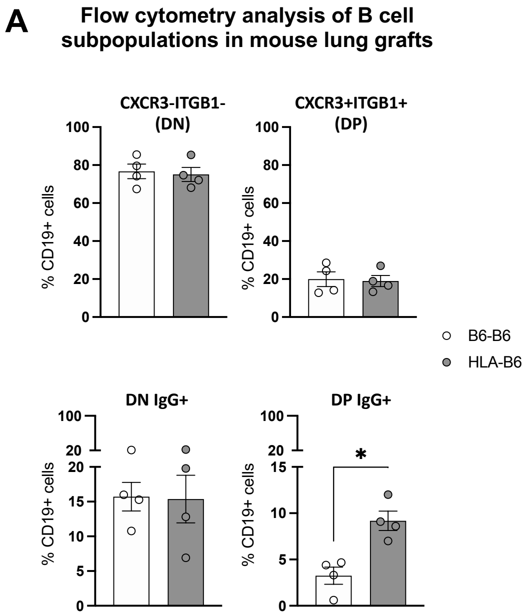

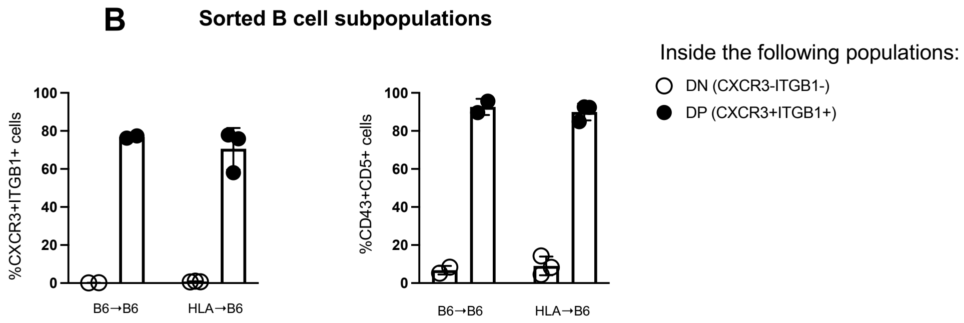

Supplementary Figure 5

Comparison of murine lung B cell clusters to B cell subsets of GSE32372 GEO database

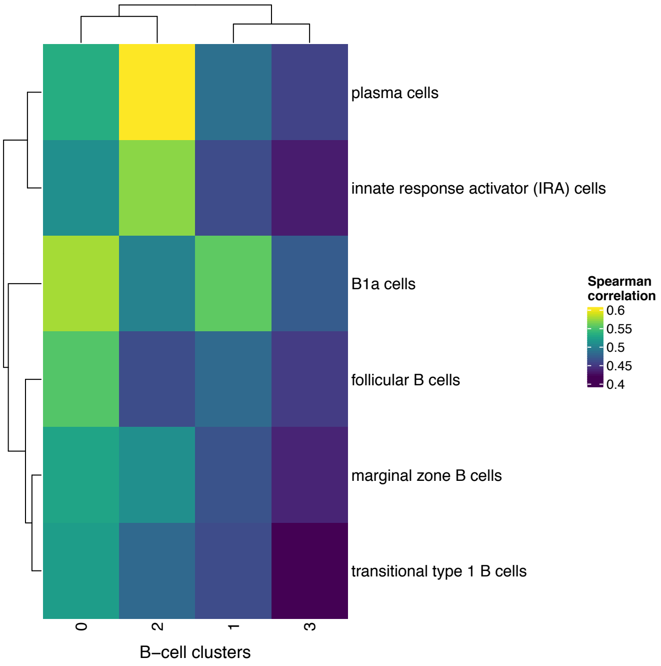

Supplementary Figure 6
